# Dizziness and benign paroxysmal positional vertigo among retirement home residents: a cross-sectional descriptive and interventional study

**DOI:** 10.1186/s12877-022-02818-w

**Published:** 2022-02-12

**Authors:** Rainer Müller, Peter Zangger, Dominik Straumann, Stefan Y. Bögli

**Affiliations:** 1 Department of Neurology, University Hospital Zurich, Zurich, Switzerland; 2 University of Zurich, Zurich, Switzerland; 3 Clinical Neuroscience Center, University Hospital Zurich, Zurich, Switzerland

**Keywords:** vertigo, Dizziness, Benign paroxysmal positional vertigo, Epidemiology, Retirement home

## Abstract

**Background:**

The prevalence of dizziness increases with age. We aimed to determine the point prevalence of dizziness and, in particular, of benign paroxysmal positional vertigo (BPPV) among retirement home residents. Furthermore, we aimed to evaluate the efficacy of a 2-axis turntable based BPPV treatment.

**Methods:**

We contacted all large retirement homes in or around the city of Zurich (Switzerland). 10 retirement homes (with a total of 536 residents) agreed to participate in this study. 83 rejected inquiries by residents led to a potential study population of 453 residents. After a structured interview evaluating the presence and characteristics of dizziness, all willing patients were tested for positional vertigo and nystagmus on a portable and manually operated 2-axis turntable that was transported to the retirement home. Testing consisted of the Dix-Hallpike and supine roll maneuvers to both sides. Participants were immediately treated with the appropriate liberation maneuver whenever BPPV was diagnosed. Otherwise, taking the resident’s medical history, a neuro-otological bedside examination, and a review of the available medical documentation was used to identify other causes of dizziness.

**Results:**

Out of the 453 residents, 75 (16.6%; average age: 87.0 years; 68% female) were suffering from dizziness presently or in the recent past and gave their consent to participate in this study. Among the participants tested on the turntable (*n* = 71), BPPV was present in 11.3% (point prevalence). Time-related properties, triggering factors and qualitative attributes of vertigo or dizziness were not significantly different between the dizzy participants with and those without BPPV. In all BPPV patients, appropriate liberation maneuvers were successful.

**Conclusions:**

BPPV could be demonstrated in about one tenth of retirement home residents with dizziness or recent dizziness. Such point prevalence of BPPV translates to a much higher yearly prevalence if one assumes that BPPV is not present on every day. Our finding suggests that retirement home residents suffering from dizziness should be regularly tested for BPPV and treated with appropriate liberation maneuvers, ideally on turntable to reduce strain.

**Trial registration:**

ClinicalTrials.gov Identifier NCT03643354.

## Background

Dizziness is one of the most common medical problems, especially in the older population. Various studies [1–3] indicate a steady increase of dizziness with age. The prevalence of dizziness in people who exceeded the age of 65 is around 30% [2, 3] and rises to almost 50% [3] in those older than 85. In particular, among nursing home residents prevalence of dizziness has been found to be up to almost 45% (peaking between 80 and 90 years of age) [4].

Etiologies of dizziness include peripheral and central vestibular, cardio-vascular, ocular motor, visual, and somatosensory disorders. In most cases, a well-focused patient history, a small set of neuro-otological bedside tests and a orthostatic evaluation suffice for identifying the primary causes of dizziness [5]. Neuro-otological bedside tests [6] should always include diagnostic maneuvers for benign paroxysmal positional vertigo (BPPV) [7].

Among older people the most frequent origin of dizziness or vertigo is BPPV [8], accounting for about one third of diagnoses [1, 9]. Typically, the first episode of BPPV occurs between 49 and 60 years [8, 10–12]. Despite the severe impact of BPPV on the quality of older individual’s lives [1, 13, 14] and the associated higher risk of falls [3, 15–17], BPPV often remains unrecognized [9, 18] and therefore the cause of dizziness remains unclear [9, 19]. Successful therapeutic maneuvers in older patients with BPPV lead to a significant reduction of falls [16, 17], thus the underdiagnosing of BPPV needs to be urgently addressed.

Pathophysiologically, there are a variety of reasons why the prevalence of BPPV increases with age. Both osteoporosis as well as vitamin D deficiency have been found to be more prevalent in patients with BPPV [20, 21]. Both lead to a disruption of the calcium metabolism and have thus been implicated in easing fragmentation or displacement of otoconia [22]. Prolonged recumbancy (e.g. due to impaired mobility) might also facilitate detachment of otoconia [23]. Lastly, older patients have higher recurrence rates of BPPV even after successful repositioning [24].

In otherwise healthy individuals, BPPV can easily be diagnosed with the provocation maneuvers, e.g., Dix-Hallpike and supine roll maneuvers [7]. However, in frail patients – who are mostly older individuals – the correct performance of the maneuvers at the bedside might be hampered, amongst other reasons, by stiffness, pain, and angst [25]. These factors may also lead to hesitations by the treating physicians. Hence, BPPV in older dizzy patients is probably more prevalent than previously thought. The highly effective therapeutic maneuvers, e.g., Epley, Sémont and Gufoni liberation maneuvers, put even more strain on frail patients when performed at the bedside. Thus, to omit neck and head movements relative to the trunk, head rotations relative to gravity, as required by both the provocation and liberation maneuvers, they are best accomplished in frail patients by whole-body rotations on a turntable [26]. Furthermore, liberation maneuvers performed on mechanical turntables might be more successful than classical manually performed maneuvers [27, 28]. The effectiveness of liberation maneuvers using mechanical turntables in retirement home residents has, to our best of knowledge, not been reported yet.

Using a manually driven 2-axis turntable we aimed to determine the point prevalence of BPPV in older patients suffering from dizziness at the day of testing or in the recent past. The turntable is portable, which allowed us to do the testing at the patients’ retirement homes. We also monitored the effectiveness of liberation maneuvers that were performed whenever BPPV was diagnosed. Secondarily, we aimed to evaluate whether there were significant differences in the presentation of symptoms between patients with BPPV and patients with dizziness of other origins.

## Methods

The study protocol was approved by the local ethics committee (cantonal ethics commission Zurich, BASEC-Nr. 2018–02017) and was in accordance with the ethical standards laid down in the 2013 Declaration of Helsinki for research involving human subjects. Informed consent was obtained from each subject — for both study participation and for publication of identifying images in an online open-access publication. The clinical trial data and the results are reported on clinicaltrials.gov (Identifier: NCT03643354).

### Recruiting process

**Fig. 1:**
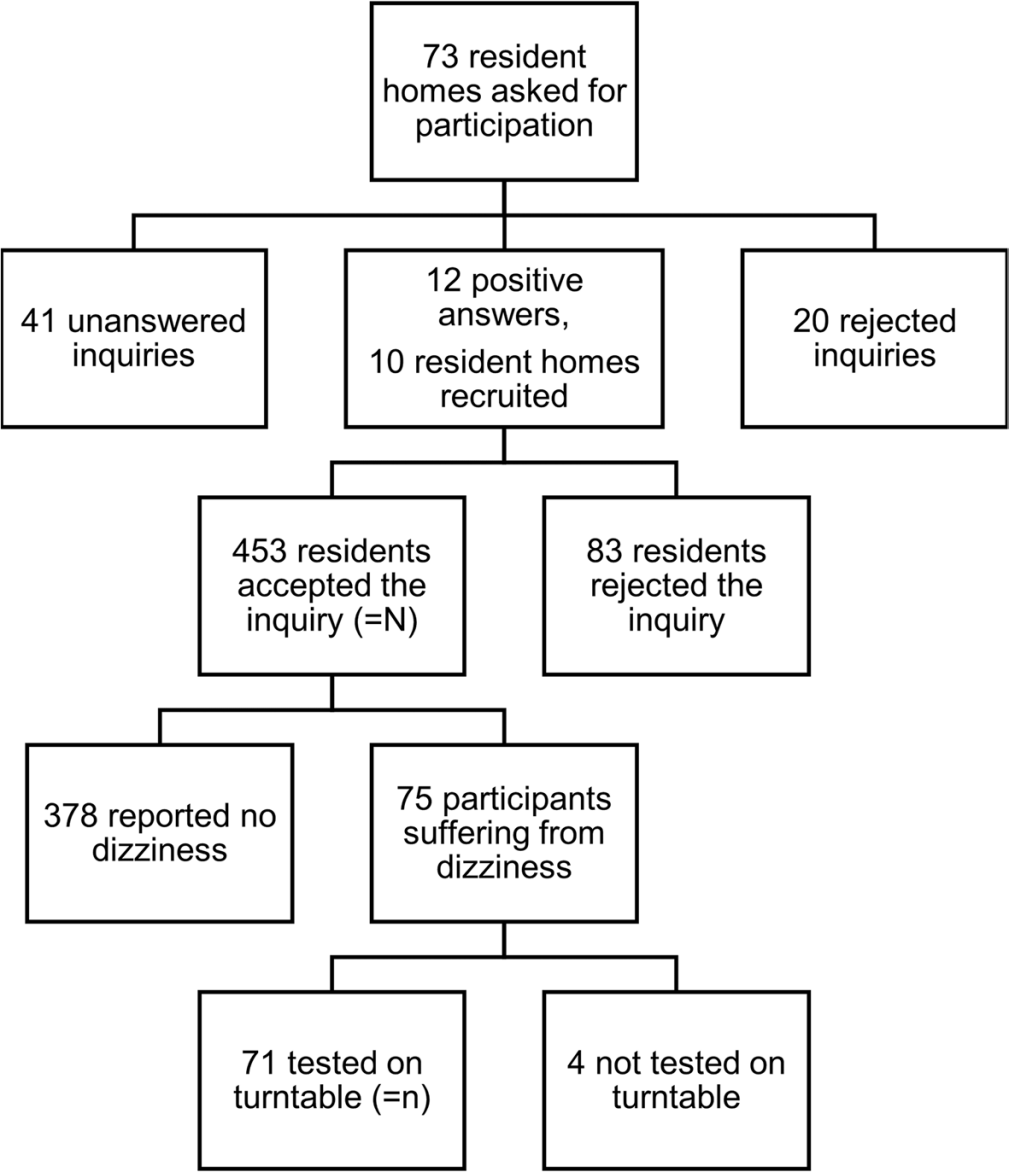
Recruitment Process

**Fig. 2:**
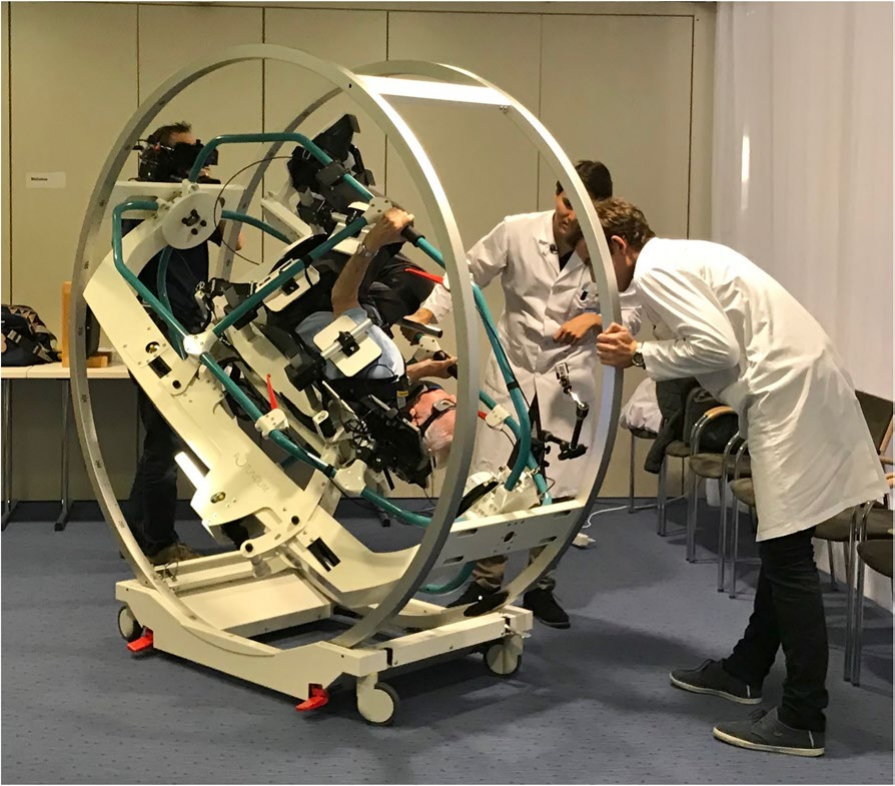
The “Rotundum” turntable in action

This study on older individuals living in retirement homes was performed at 10 institutions in the Canton of Zürich, Switzerland, between 2018 and 2020. During the recruiting process (Fig. 1), we contacted the management of 73 retirement homes and asked whether we could visit their facilities with our “Schwindelbus” containing the the “Rotundum” turntable (Fig. 2). This van would transport the portable 2-axis turntable to the respective retirement home and set up in a larger area within the facility, typically a recreation room. There, any resident with present or recent dizziness would be offered diagnostic and possibly therapeutic BPPV maneuvers on the turntable. This service would be free of charge. The anonymized data would be analyzed scientifically with ethical permission. Surprisingly, the managements of 63 retirement homes were not interested or did not respond, despite repeated enquiry. Reasons for disinterest included the rejection to participate in research, the notion that dizziness was not a major problem among the residents, and the fact that the caregivers were too busy.

The managements of 10 retirement homes, however, agreed to participate. All retirement home residents (536 residents) were informed of the upcoming visit by the “Schwindelbus” and about the opportunity to receive specialist advice on the topic of dizziness.

All residents who agreed to inclusion were evaluated for the presence of dizziness. Residents who experienced dizziness presently or in the recent past (defined as having occurred at least once within the last 3 months) were interviewed by one of the co-authors (R.M.) based on a questionnaire covering major aspects of their dizziness and its impact on their daily life. Furthermore, age and gender were noted. The questionnaire (translated from German to English) used can be found as a supplement of this paper. Dizziness was enquired using the German word “Schwindel”, which encompasses a variety of symptoms including subjective imbalance, illusion of motion (of either the body or the surrounding), as well as light-headedness, commonly appearing together with different degrees of nausea. Different aspects of dizziness including persistence (i.e. chronic persistent versus recurrent attacks), duration (i.e. up to few minutes versus hours to days), number (i.e. multiple times daily versus once daily down to less than once per month), interictal symptoms (i.e. minor dizziness versus no dizziness), presence of triggering factors (i.e. lying down, standing up quickly, turning in bed, reclination of the head), characteristic of dizziness (turning versus swaying), as well as dizziness-induced nausea or vomiting were inquired. Impairment of quality of life was queried both on a completely subjective level (i.e. does the dizziness impair your quality of life) as well as depending on different daily activities (including activities performed while lying, sitting or standing). Residents who were unable to answer the structured interview questions due to cognitive impairment were excluded from the study. Altogether, 75 out of 453 residents addressed suffered from dizziness or vertigo (Fig. 1).

### “Schwindelbus” visit

Within 1 week after the structured interview, participants were examined on a manually driven 2-axis turntable (Rotundum R1, prolim GmbH). Of the 75 individuals 4 could not be tested on the turntable, as they were not able to be present on the day of examination or were afraid of the procedure. 71 residents (= n) underwent diagnostic BPPV provocation maneuvers on the turntable. These were the Dix-Hallpike maneuver [29] (whole-body rotation 30 deg below the horizontal) on both sides and the supine roll maneuver [30] (by 90 deg) on both sides. Eye movements were recorded and monitored for canal-specific positional nystagmus. Participants were asked to immediately report any sensation of dizziness or vertigo. If the diagnosis of BPPV was established by the pathognomonic nystagmus associated with rotational vertigo, the appropriate liberation maneuvers were performed. The liberation maneuver was performed maximally 2 times on a single day. As many patients suffer from dizziness immediately after repositioning maneuvers, effectiveness was evaluated clinically based on the absence of nystagmus in the subsequent provocation maneuvers (performed immediately after the liberation maneuvers).

Participants in whom BPPV was excluded, underwent a short neuro-otological bedside examination (including ocular motor stability, head impulse test, Romberg testing on solid ground and on foam, bimalleolar vibration sense). Furthermore, a short patient’s history was taken, and a review of the available medical documentation was used to identify other causes of their dizziness.

### Follow-up visit

The participants diagnosed with BPPV were approached a third time after 1 to 2 weeks. They were interviewed on the treatments effect as evaluated by the subjective effect on their quality of life. At this follow-up visit, we did not have the opportunity to examine the patients clinically or on the turntable.

### Data analysis

Data analysis was performed using IBM SPSS Statistics 26 (IBM, Armonk, NY, USA). Descriptive statistics are reported as counts/percentages or mean ± standard deviation as appropriate. Categorical variables are compared using the Fisher’s exact test. The yearly estimated prevalence *P*_*y*_ of a disease was calculated by:

$${P}_y=\frac{365\ }{D}x\ {P}_d$$

where *D* is the estimated duration of a disease (in days) over the period of 1 year and *P*_*d*_ point prevalence of the same disease.

### Data availability statement

Anonymized data used in this study are available upon reasonable request from Dr. S.Y. Bögli.

## Results

**Fig. 3:**
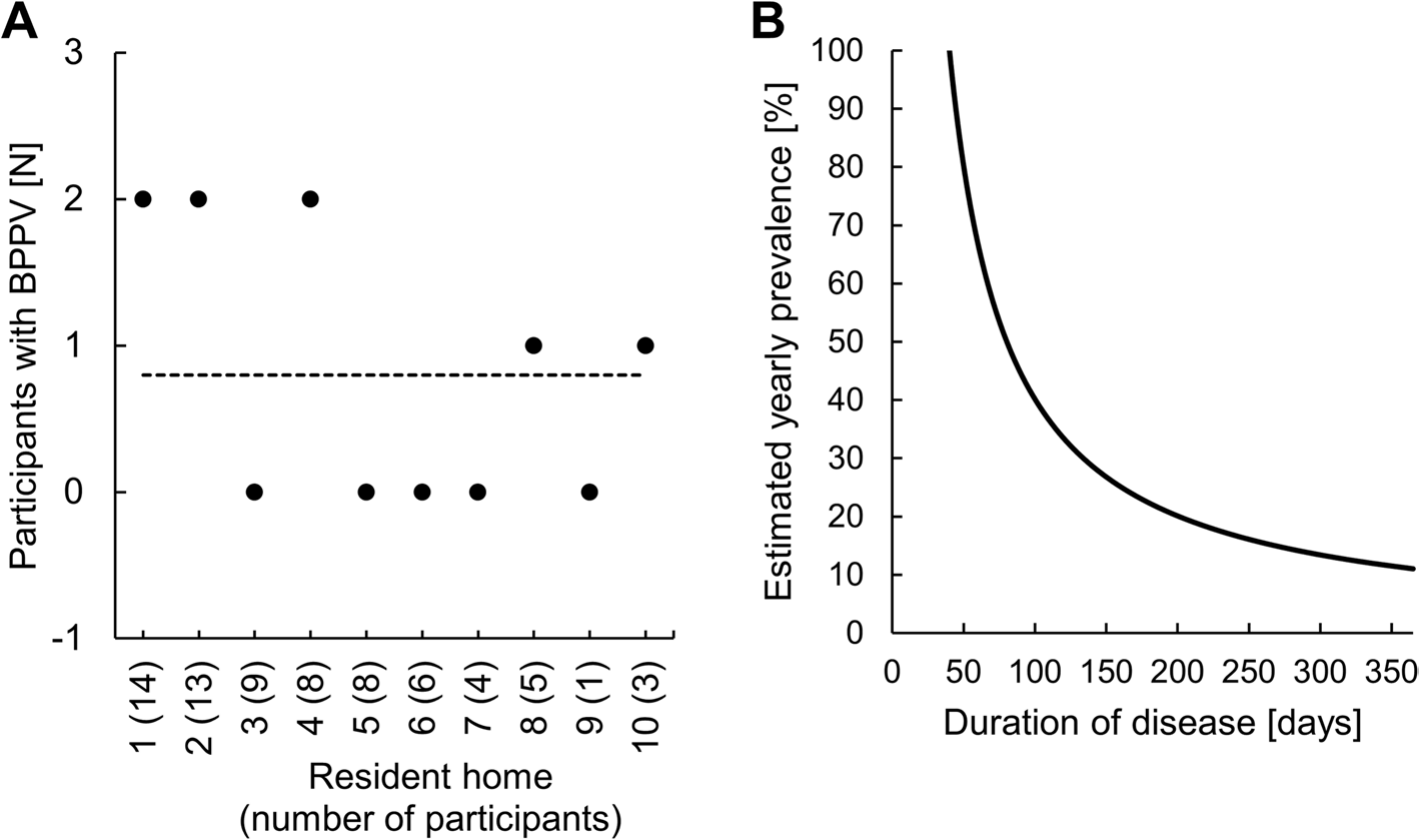
Point prevalence and estimated yearly prevalence of BPPV. Panel A shows the number of residents per resident home that were diagnosed with BPPV. The dotted line corresponds to the average. Based on the found BPPV point prevalence of 11.3%, panel B depicts estimated BPPV yearly prevalence as a function of BPPV duration (in days) over the period of 1 year (formula given in Methods)

Present or recent dizziness was reported by 75 (16.6%) individuals among the potential study population of 453 residents at the 10 retirement homes in or around the city of Zurich. 68% were female (mean age: 87.7 ± 5.6 years) and 32% male (mean age: 85.5 ± 7.8). Of the 71 participants who underwent diagnostic rotations on the manual 2-axis turntable, 8 (= 11.3%) showed the positional nystagmus and rotational vertigo typical of BPPV in one of the provocation maneuvers. The prevalence as well as the estimated yearly prevalence are shown in Fig. 3. In all cases, the Dix-Hallpike maneuver was positive on the left or right side indicating canalolithiasis of the respective posterior canal. If all residents (*N* = 453) correctly indicated whether they suffered from dizziness presently or in the recent past, the point prevalence of BPPV would amount to 1.8%. Based on the questionnaire and the subsequent short neuro-otological bedside examination, most of the remaining 61 (= 86%) participants could be assigned to defined diagnoses among which orthostatic dizziness was the most frequent (Table 1).

**Table 1:** Clinical diagnoses

Primary diagnosis	Number of patients	Percentage of patients
**Orthostatic dizziness**	19	26,8%
**Multifactorial dizziness**	17	23,9%
**BPPV**	8	11,3%
**Proprioceptive dizziness**	3	4,2%
**Vestibular migraine**	3	4,2%
**Cardiogenic dizziness**	2	2,8%
**Unilateral vestibulopathy**	2	2,8%
**Central vertigo**	2	2,8%
**Status post BPPV**	2	2,8%
**Bilateral vestibulopathy**	1	1,4%
**Psychogenic vertigo**	1	1,4%
**Meniere’s disease**	1	1,4%
**Unclear cause**	10	14,1%

We asked whether symptoms of dizziness in the participants with BPPV would differ from those with other diagnoses. Time-related properties (e.g., chronic vs. episodic, frequency, duration), triggering factors (e.g., turning in bed) and qualitative attributes (e.g., spinning vertigo, unsteadiness) were all not significantly different between the two groups of participants (Table 2).

**Table 2:** Univariate analysis of symptoms associated with BPPV

		No. of positive responses		Fisher exact test (p)
		Without BPPV (*n* = 63)	With BPPV (*n* = 8)	
Constant dizziness or dizzy spells	Dizzy spells	54	8	0.59
	Constant dizziness	8	0	
	Not able to answer	1	0	
Duration of dizzy spells	Seconds to minutes	38	6	0.32
	Hours to days	14	0	
	Not able to answer	3	2	
Frequency of dizzy spells	Daily	30	5	1.00
	Less often	22	3	
	Not able to answer	3	0	
Between attacks	Free of dizziness	44	8	0.33
	Less dizzy	10	0	
	Not able to answer	1	0	
Precipitating factor: Backward tilt of the head	Yes	18	5	0.13
	No	36	3	
	Not able to answer	9	0	
Precipitating factor: Turning in bed	Yes	10	2	0.27
	No	43	3	
	Not able to answer	10	3	
Precipitating factor: Rising from supine or sitting position	Yes	48	8	0.34
	No	11	0	
	Not able to answer	4	0	
Characterization of dizziness	Spinning sensation	26	5	0.28
	Staggering or swaying vertigo	17	2	1.00
	Light headedness	13	0	0.34
	Unstableness	5	1	0.53
	General sense of weakness	2	0	1.00
	Not able to answer	2	0	
Nausea or vomiting	Yes	10	2	0.62
	No	51	6	
	Not able to answer	2	0	
Deterioration in the quality of life	Yes	35	6	0.47
	No	24	2	
	Not able to answer	4	0	

Participants, in whom BPPV was diagnosed on the manual 2-axis turntable, were immediately treated with the Epley liberation maneuver [31], whereby the longitudinal body axis was 30 deg below the earth-horizontal and the rotations were performed in 45-deg-steps. In 7 patients a single Epley maneuver to liberate the affected semicircular canal was sufficient with no positional nystagmus and vertigo in the subsequent Dix-Hallpike maneuver. The Epley maneuver had to be repeated in 1 patient to reach absence of positional nystagmus und vertigo.

Participants with diagnosed and treated BPPV were asked about their dizziness 1 to 2 weeks after the “Schwindelbus” visit. 2 individuals were free of dizziness. The other 6 indicated that they were still suffering from dizziness. Of these participants, one reported a decrease of dizzy spells in both frequency and severity, and one an improvement in quality of life.

## Discussion

We visited 10 retirement homes to determine the point prevalence of BPPV among the residents who suffered from dizziness presently or in the recent past. In older patients, diagnostic bedside maneuvers for BPPV (Dix-Hallpike and supine roll maneuvers) are often compromised due to stiffness, pain, and angst, especially among frail patients. For this reason we used a manual 2-axis turntable that was brought by a van (“Schwindelbus”) to each resident home where we tested the dizzy residents. By using such a turntable, movements of the head relative to the trunk can be minimized, which considerably reduces the strain on spine, shoulder and trunk during BPPV maneuvers [26].

Among the retirement home residents suffering from dizziness, BPPV was present in 11.3% at the time of turntable testing. Figure 3 demonstrates the theoretical relation between yearly prevalence and the number of symptomatic days. BPPV is commonly a transient, but frequently recurrent disease. It can only be diagnosed when it is symptomatic. Assuming that BPPV is symptomatic every day of the year, the yearly prevalence would coincide with the point prevalence, in this case 11.3%. On the other hand, assuming (for example) that BPPV among dizzy patients is only symptomatic during 60 days over a year, the yearly prevalence would have to be much higher (i.e., 69%) to allow for a point prevalence of 11.3%. There is no way of knowing the number of symptomatic days without testing participants several times during a year, which was beyond the scope of this study. In a representative neuro-otological survey of the general adult population, 8.0% of patients with moderate or severe dizziness had typical symptoms of BPPV [10], which is somewhat lower than the percentage we found by the actual testing of retirement home residents.

The discrepancy between the BPPV point prevalence of 11.3% in dizzy retirement home residents, as found in our study, and the BPPV prevalence of 27.6–37.6% in older patients evaluated at dizziness units [1, 9] is probably due to selection, as these studies were performed in hospitals compared to our study that was performed within retirement homes. Only older individuals, in whom the level of dizziness is high enough and outweighs the strain of a transport to the hospital, are evaluated at dizziness units.

Over the potential study population of 453 of the 10 retirement homes the point prevalence of BPPV amounted to 1.8%, which is even higher as the yearly prevalence (1.6%) in the general population [10]. Again, the point prevalence of BPPV is most likely considerably lower than the yearly prevalence, which indicates a much higher burden of BPPV on the older population. Furthermore, there is a selection bias within our study, as some dizzy patients might have either not been able to answer the questionnaire (and were thus excluded) or were not willing to participate in the study lowering the point prevalence found. Therefore, the percentage of residents who reported present or recent dizziness (16.6%) needs to be taken with great caution. Structured interviews would need to be conducted among all retirement home residents by investigators with high interrater reliability to determine a reliable percentage of dizzy patients in the population.

All participants with BPPV were successfully treated on the turntable as quantified by the absence of nystagmus in the subsequent control maneuvers. Interesting from a clinical perspective was that time-related properties, triggering factors and qualitative attributes of vertigo or dizziness were not significantly different between the dizzy residents with and those without BPPV. This finding supports the approach to perform diagnostic BPPV maneuvers on every older individual with dizziness irrespective of the presence of a “characteristic” symptom constellation. In spite of the absence of nystagmus in the control maneuvers, only 25% of residents treated on the turntable were free of dizziness at the follow-up visit. In concordance with the lack of distinct symptoms differentiating residents with and without BPPV, it can be expected that the other residents treated for BPPV were also suffering from other types of dizziness. However, as the study was concluded after the follow-up visit (that did not include a further evaluation of their dizziness origin), this can only be hypothesized.

We conclude that diagnostic BPPV maneuvers are positive in about one tenth of retirement home residents with dizziness or recent dizziness. As BPPV is not always symptomatic, the yearly prevalence is probably higher. In this population, there is a lack of symptoms that clearly differentiate between those with and those without BPPV. This emphasizes the need for a low-threshold implementation of provocation maneuvers. Retirement home residents suffering from dizziness should regularly be tested for BPPV even in the absence of characteristic symptoms. Diagnostic and therapeutic BPPV maneuvers should ideally be performed on a turntable to reduce strain, improve diagnosis and optimize therapy.

## Data Availability

The datasets used and/or analyzed during the current study are available from the corresponding author on reasonable request.

## Abbreviations

BPPV: Benign paroxysmal positional vertigo
